# Novel High-Molecular Weight Fucosylated Milk Oligosaccharides Identified in Dairy Streams

**DOI:** 10.1371/journal.pone.0096040

**Published:** 2014-05-08

**Authors:** Raj Mehra, Daniela Barile, Mariarosaria Marotta, Carlito B. Lebrilla, Caroline Chu, J. Bruce German

**Affiliations:** 1 Teagasc Food Research Centre, Moorepark, Fermoy, Co. Cork, Ireland; 2 Department of Food Science and Technology, University of California, Davis, Davis, California, United States of America; 3 Department of Chemistry, University of California, Davis, California, United States of America; 4 Foods for Health Institute, University of California, Davis, California, United States of America; Oxford University, United Kingdom

## Abstract

Oligosaccharides are the third largest component in human milk. This abundance is remarkable because oligosaccharides are not digestible by the newborn, and yet they have been conserved and amplified during evolution. In addition to encouraging the growth of a protective microbiota dominated by bifidobacteria, oligosaccharides have anti-infective activity, preventing pathogens from binding to intestinal cells. Although it would be advantageous adding these valuable molecules to infant milk formula, the technologies to reproduce the variety and complexity of human milk oligosaccharides by enzymatic/organic synthesis are not yet mature. Consequently, there is an enormous interest in alternative sources of these valuable oligosaccharides. Recent research has demonstrated that bovine milk and whey permeate also contain oligosaccharides. Thus, a thorough characterization of oligosaccharides in bovine dairy streams is an important step towards fully assessing their specific functionalities. In this study, bovine milk oligosaccharides (BMOs) were concentrated by membrane filtration from a readily available dairy stream called “mother liquor”, and analyzed by high accuracy MALDI FT-ICR mass spectrometry. The combination of HPLC and accurate mass spectrometry allowed the identification of ideal processing conditions leading to the production of Kg amount of BMO enriched powders. Among the BMOs identified, 18 have high-molecular weight and corresponded in size to the most abundant oligosaccharides present in human milk. Notably 6 oligosaccharides contained fucose, a sugar monomer that is highly abundant in human milk, but is rarely observed in bovine milk. This work shows that dairy streams represent a potential source of complex milk oligosaccharides for commercial development of unique dairy ingredients in functional foods that reproduce the benefits of human milk.

## Introduction

Human milk oligosaccharides (HMOs) are the third largest fraction in human milk after lactose and fat, with concentrations of up to 25 g/L in colostrum, and 10–15 g/L in mature milk, being about 20-fold greater than in bovine milk [1], [2]. The high concentrations of human milk oligosaccharides combined with the fact that they are indigestible to humans have raised the question of the function of these glycans. It is now widely accepted that HMOs exert several biological activities that are firmly dependent on their individual chemical structures. The core structures are mainly based on lactose, which is modified by enzymatic addition of specific neutral monosaccharides such as *N*-acetylglucosamine or *N*-acetylgalactosamine (HexNAc), galactose or glucose (Hex) and fucose or deoxyhexose (Fuc) (neutral oligosaccharides), or acidic components such as *N*-acetylneuraminic acid also known as sialic acid (acidic oligosaccharides) [3]. Various enzymes in the mammary gland combine these monomers into diverse structures; however, the precise biosynthetic steps leading to oligosaccharides are still unknown. Systematic characterization of HMOs started in the 1950s, yet analysis remains a challenging task due to the number of unique structures and their overall complexity. At present, more than 200 potential structures have been identified in human milk [4]–[6]. Neutral oligosaccharides containing *N*-acetyl-glucosamine (GlcNAc) are the most relevant factors for the development of the intestinal flora typical of breast-fed infants [2], [7]. High concentrations of oligosaccharides in milk correlate with a higher diversity of *Bifidobacterium* species [8]. Interestingly, activity of many enteric pathogens are blocked by HMOs, and for several pathogens fucosylated and sialylated oligosaccharides proved to be the active components [9]–[13]. HMOs block the ability of a pathogen to bind to, and infect its host cell, making the host niche of the pathogen effectively unavailable. HMOs have been consumed by infants for millennia, and the appearance of pathogenic strains resistant to these oligosaccharides has never been reported.

As it becomes more evident that HMOs play a key role in promoting and maintaining good health in breastfed infants, it would be advantageous also to provide HMOs not only to breastfed infants, but also to individuals of all ages through food supplements. However, it is currently not possible to replicate the complex oligosaccharides structures by synthetic methods. Several food companies are actively investigating methods to produce synthetic oligosaccharides, but only the smallest HMOs have been reproduced with success and they are not representative of the complexity and variety of the HMOs. Since HMOs are not available in large quantities, there is an enormous interest in identifying alternative sources of these valuable molecules.

Recent research from our group and others has demonstrated that bovine milk and dairy streams (whey streams) contain several oligosaccharides, with a number of them structurally similar to those found in human milk and with some structures common to both milks [14]–[18]. As chemical structure of oligosaccharides influences their biological activities, it is expected that oligosaccharides from both human and bovine milks would have similar physiological effects. Consequently, there is interest in adding these valuable molecules to infant formulas to better match the nutritional value of these products to breast milk. Indeed, oligosaccharides are not commercially available from human milk. Additionally, given the importance of the dairy industry globally and the well established technologies available within it, bovine milk and whey streams are being considered as a key source of oligosaccharides. However, concentration of bovine milk oligosaccharides (BMOs) is low compared to the one of HMOs. In this respect, an essential step to increase our capability to characterize and utilize oligosaccharides from whey streams is to increase their total abundance. In this study, BMOs were concentrated by membrane filtration from a readily available whey stream, and analyzed by high accuracy mass spectrometry.

Whey is the liquid part of milk that separates from the curd during cheese production. Whey is rich in lactose and protein, but its high biochemical and chemical oxygen demand make it expensive to dispose of within environmental regulations. Whey is a valuable dairy stream for extraction of useful proteins in the form whey protein concentrates by ultrafiltration (UF) [14], [15]. Whey UF permeate is either disposed of at a cost to the whey processor or used to produce food grade lactose by crystallization. The liquid separated from lactose crystals, known as mother liquor, is usually disposed of to sewage plants or sold as animal feed. Because mother liquor is already virtually protein-free and most of the lactose has already been removed, oligosaccharides can be purified by filtration, which allows retention of oligosaccharides and removes lactose and salts. Such mother liquor enriched in oligosaccharides would facilitate commercial use of these bio-functional ingredients for the food, beverage, and baby food industries, add value to the waste streams of dairy processing, and also reduce waste disposal costs for the dairy industry.

The present study employed large-scale centrifugation and membrane filtration technology to produce powders enriched in BMOs using mother liquor as a low-cost starting raw material. Mother liquor was processed employing membranes that fractionated and concentrated BMOs from the other molecules. Mass spectrometry analysis of oligosaccharides derived from this process revealed numerous BMOs including eighteen high-molecular weight species never previously described. Importantly, some of these novel BMOs contained fucose, a key component of HMOs.

## Materials and Methods

Mother liquor used in this study was purchased from Glanbia plc, Ireland, and transferred refrigerated to Moorepark Technology Limited, a pilot plant facility of Teagasc Food Research Centre, Moorepark Fermoy, Co. Cork, Ireland.

### Clarification of Mother Liquor by Centrifugation and Microfiltration

The initial clarification of mother liquor (2000 kg) to remove large insoluble material was carried out at 24°C using a pilot plant-scale Westfalia centrifugal clarifier (Model KNA 3) with a flow capacity of 1000 L/h (GEA Westfalia Separator GmbH, Germany).

The supernatant fraction obtained from initial clarification was microfiltered through 0.1 mm molecular weight cut-off (MWCO) membranes using a pilot scale Tetra Alcross MSF19 microfiltration plant (Tetra Pak Filtration Systems A/S, Aarhus, Denmark). The plant has 2 loops, each equipped with tubular ceramic multichannel membranes (19 filter channels, 102 cm long, and 3 mm channel diameter) enclosed in acid-proof stainless housings. The total membrane surface area of this plant was 12.5 m^2^. Membranes were cleaned according to the manufacturer’s instructions before and after use. Filtration was performed at 20°C at the following pressures: retentate inlet, 29.3 kPa; retentate outlet, 11.7 kPa; permeate inlet, 24.1 kPa, and permeate outlet, 15.8 kPa. This step removed the residual insoluble material and bacteria from the mother liquor, leaving soluble mineral salts, lactose and oligosaccharides in the microfiltrate.

### Fractionation and Enrichment of Milk Oligosaccharides by Membrane Filtration

The microfiltrate from the microfiltration step was utilized as the feed to the ultrafiltration plant for fractionation and enrichment of milk oligosaccharides. The process was carried out using six, 1-kDa MWCO, spirally wound membranes (Desal Membrane Products from GE Osmonics, Minnetonka, MN, USA) with a total membrane surface area of 30 m^2^. Ultrafiltration was carried out in batch mode at about 10°C, with inlet and outlet pressures of 551.6 kPa and 413.6 kPa, respectively. The purpose of this step was to separate and concentrate oligosaccharides from lactose and mineral salts. The oligosaccharides-rich retentate was then diafiltered with reverse osmosis water in five consecutive batch diafiltration steps to further wash out the lactose into the permeate, thus allowing further enrichment of the retained oligosaccharides. The final diafiltered retentate was concentrated by evaporation at 50°C and spray dried with inlet and outlet temperatures of 120°C and 80°C, respectively. This process yielded 2.8 kg of bovine milk oligosaccharides-rich powder. Lactose and sialyllactose, being the smallest oligosaccharides present in milk and whey streams for which commercial standard exist, were chosen as markers of oligosaccharide retention and enrichment during processing.

### Quantification of Lactose and Sialyllactose by HPLC

Whey permeate, mother liquor, diafiltered oligosaccharides-enriched retentate and milk oligosaccharides-rich powder, appropriately diluted in water, were analyzed for quantification of lactose and sialyllactose.

Lactose was quantified by HPLC using an HPX-87C Carbohydrate column (300×7.8 mm) (Aminex, Bio-Rad, UK) and a refractive index detector. The elution was obtained in isocratic conditions using 9 mM sulphuric acid for 30 min.

Both sialyllactose isomers (3′-sialyllactose and 6′-sialyllactose) were quantified by High pH Anion Exchange Chromatography with Pulsed Amperometric Detection, using a CarboPac PA 100 (250×4 mm) connected to a CarboPac PA 100 guard column and equipped with an electrochemical detector (Dionex Corporation, Sunnyvale, CA). Elution was carried out with the following gradient: 100 mM NaOH (Eluent A) and 100 mM NaOH, 500 mM NaAc (Eluent B) (t = 0–3 min 95% Eluent A; t = 3–13 min 88% Eluent A; t = 13–30 min 50% Eluent A; t = 30–45 min equilibrated at 95% Eluent A).

### Identification of Oligosaccharides by Fourier Transform-Ion Cyclotron Resonance Mass Spectrometry (FT-ICR MS)

Samples collected during the process were purified by solid-phase extraction as previously reported to isolate oligosaccharides prior to mass spectrometry analysis [19]. Analysis of oligosaccharides was performed using a ProMALDI-FT-ICR mass spectrometer (IonSpec, Lake Forest, CA) equipped with a 7.0 Tesla superconducting magnet, hexapole ion accumulation and fitted with a 355-nm pulsed Nd:YAG laser. Samples were crystallized using 2,5-dihydroxybenzoic acid as matrix (5 mg/100 *µ*L in a solution of 50% acetonitrile/50% water (v/v)). The solution of oligosaccharides (1 µL) was applied to the MALDI probe followed by addition of 0.01 M NaCl (0.5 µL) and the matrix solution (1**µL). Sample spots were dried by a technique similar to a fast evaporation method prior to mass spectrometric analysis. Spectra were acquired in the positive-ion mode and internally calibrated using oligosaccharides from other food matrices.

Tandem Mass Spectrometry was performed to obtain compositional information using sustained off resonance irradiation (SORI) collision-induced dissociation (CID) to determine the composition and putative structure of each oligosaccharide. The precursor ion was isolated and excited to 1000 Hz of their cyclotron frequency at SORI amplitude of 2.55 V. Nitrogen gas was used as the collision gas and was pulsed in to maintain a pressure of 10^−6^ Torr.

## Results and Discussion

### Enrichment of Milk Oligosaccharides by Membrane Technology

Initial analysis of various dairy streams led to the identification and selection of mother liquor as an optimum starting material because of its higher level of oligosaccharides compared to whey permeate or skim milk permeate. By comparison to other dairy streams, mother liquor has a higher sialyllactose to lactose ratio as measured by HPLC. Typically, whey UF permeates obtained from industrial dairy production contained between 50 and 65 mg/L of sialyllactose (3′ and 6′ sialyllactose) compared with 50 g/L of lactose, whereas mother liquor samples contained about 170 mg/L of sialyllactose for a similar level of lactose (49 g/L). Thus, mother liquor was 2.5- to 3.5-fold more enriched than whey UF permeates when compared as a ratio of sialyllactose to lactose. The sialyllactose to lactose ratio (SL/L×100) in the starting mother liquor was 0.35%. Following membrane filtration and diafiltration, the diafiltered oligosaccharides-enriched retentate had a sialyllactose to lactose ratio of 14.1%. This represents a 40-fold enrichment of sialyllactose based on the SL/L ratio. On evaporating and spray drying the retentate, 2.8 kg of a powder was obtained with the following composition: 93.7% total solids, 30.3% lactose, 2.8% sialyllactose (3′+6′), 7.4% true protein, 2.0% non-protein nitrogen, 24% ash and 27.2% unidentified material (including oligosaccharides for which commercial standards do not exist).

**Figure 1:**
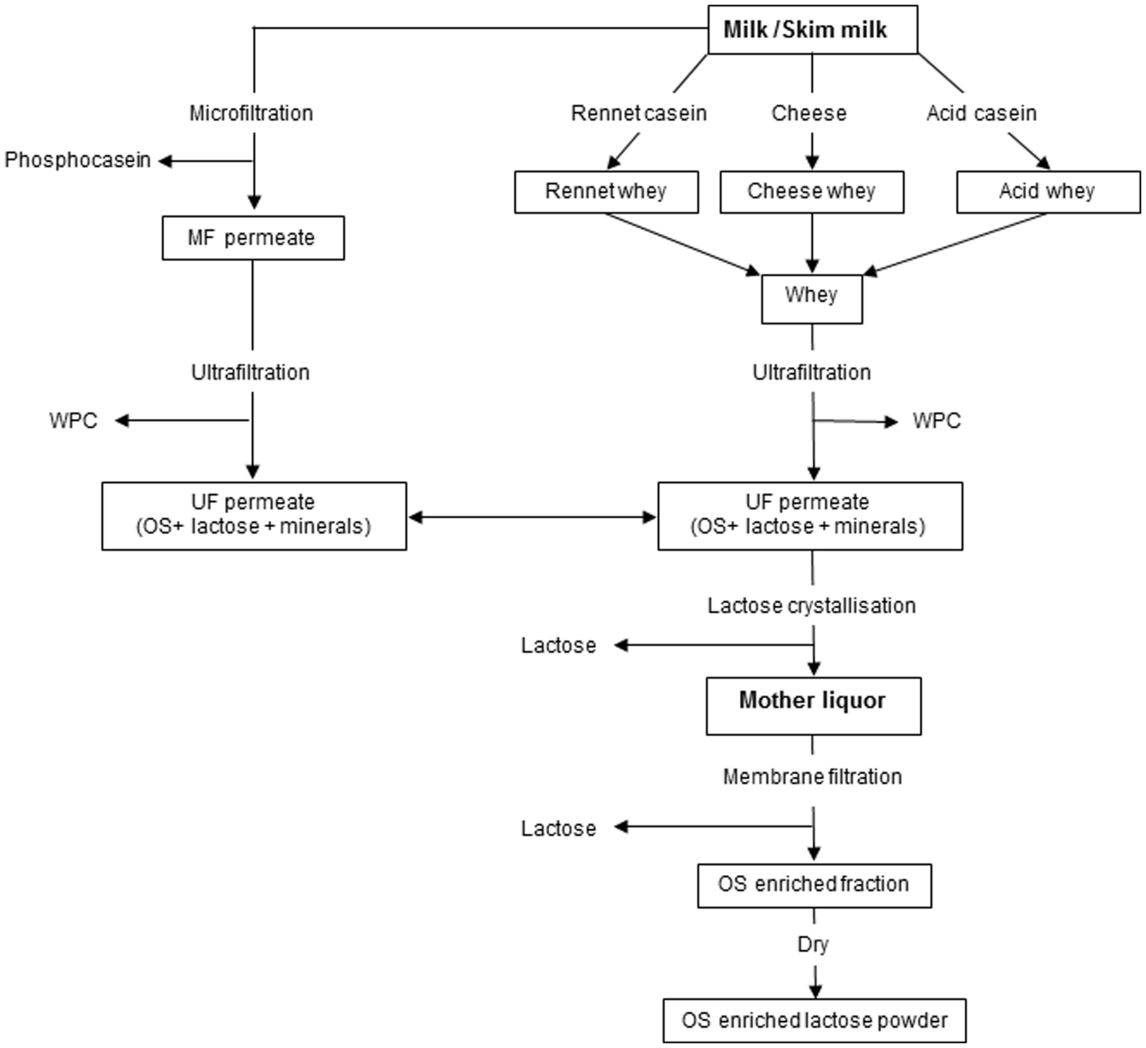
Fractionation and enrichment of oligosaccharides from mother liquor using membrane filtration technology.

**Figure 1** shows a schematic flow chart of the membrane filtration process employed for the enrichment of oligosaccharides, including the origin of industrial mother liquor from bovine milk. Earlier work carried out at pilot plant scale in Teagasc Food Research Centre Moorepark, Ireland, to evaluate various membrane types, membrane porosity (MWCO), operating conditions of pressure and feed pH suitable for such a process led to the selection of spirally-wound membrane with porosity of 1 kDa MWCO (data not shown). Other reports have also described the use of 1 kDa MWCO ceramic membrane to separate goat milk oligosaccharides at laboratory scale [20], [21].

### Identification of Oligosaccharides by FT-ICR MS

**Figure 2:**
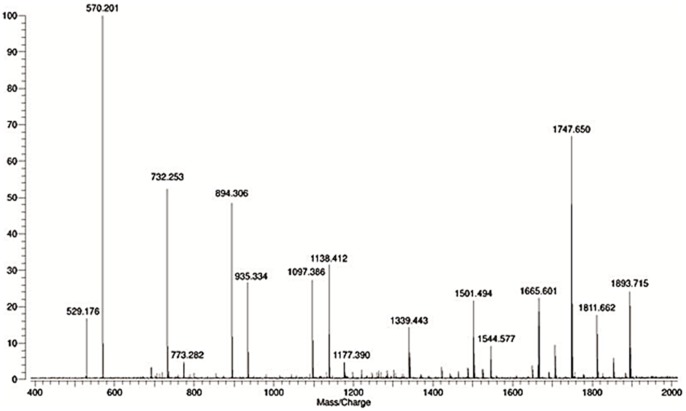
MALDI FT-ICR mass spectrum profile of the final sample mother liquor concentrated by membrane filtration. Spectrum was recorded in positive ionization mode and display all neutral oligosaccharides.

**Figure 3:**
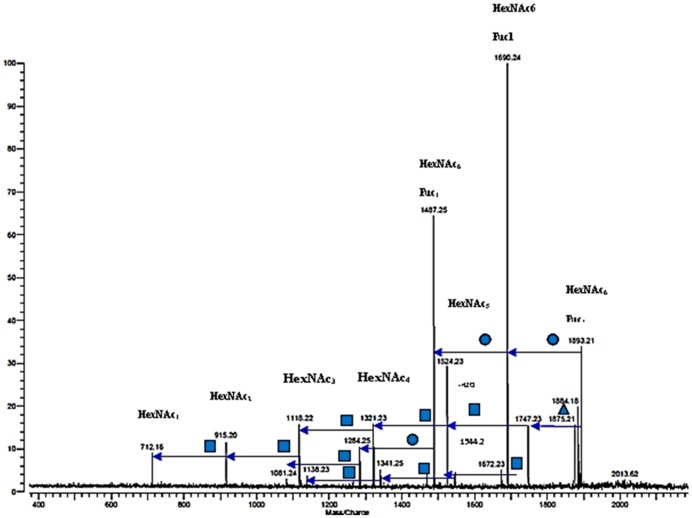
Collision-induced dissociation (CID) spectrum of peak m/z 1893 [M+Na]. The main fragmentation pattern shows the loss of one fucose (triangle) followed by the loss of 5 HexNAc (squares) leading to the residue MW 712.16 with composition 3Hex+1HexNAc. The total composition of ion MW 1893.21 3 Hex +6 HexNAc +1 Fuc (HexNAc: N-Acetylhexsamine-(GlcNAc/GalNAc); Hex: hexose; Fuc: fucose).

FT-ICR MS analysis out of the oligosaccharides-enriched powder obtained by the membrane filtration process described above resulted in the identification of 25 oligosaccharides. The instrument was ran in positive more and centered to the higher molecular weight range (up to 4000Da) to enable detecting larger neutral oligosaccharides. Oligosaccharides carrying negative charges such as those containing sialic acid produce more intense signal in negative than positive ion-detection mode of analysis because they readily deprotonate forming [M-H]-. Conversely, neutral oligosaccharides are more difficult to detect in negative mode because their ionization efficiency is lower, in fact neutral oligosaccharides have a low tendency to form [M-H]-. Therefore, to improve neutral oligosaccharides detection, we formed a metal-carbohydrate adduct using Sodium, and then performed the analyses in positive ion-detection mode, forming the adduct [M+Na]+. The spectrum of the neutral oligosaccharides found in the mother liquor powder is shown in **Figure 2**. Bovine oligosaccharides previously reported in whey [22] and milk/colostrum [16], [17], [19] were observed (i.e. those between 500–1000 m/z in Figure 2). Additionally, more complex oligosaccharides, ranging in mass from 1257 to 1891 m/z, were observed. The identified oligosaccharides are listed with their putative composition in **Table 1**. The oligosaccharides composition reveals neutral oligosaccharides containing hexoses (Hex) and *N*-acetylhexosamines (HexNAc) and six oligosaccharides containing fucose. To illustrate the mechanism of structural elucidation that led to the discovery of 6 fucosylated oligosaccharides, one example of tandem mass spectrometry by Collision-induced dissociation (CID) is provided in Figure 3. The CID spectrum of peak m/z 1893 [M+Na] shows the loss of one fucose (represented by a triangle) followed by the loss of 5 HexNAc (represented by squares) which combined with the residue with composition 3Hex+1HexNAc (MW 712) leading to a total composition of 3 Hex +6 HexNAc +1 Fucose for ion MW 1893.21.

**Table 1:** Composition of neutral oligosaccharides (reduced, sodiated masses) in mother liquor.

Masss [M+Na]^+^	Hex	HexNAc	NeuAc	NeuGc	Fuc	R/A %
529.175	3	0	0	0	0	16
570.201	2	1	0	0	0	100
732.253	3	1	0	0	0	52
773.281	2	2	0	0	0	5
894.306	4	1	0	0	0	48
935.334	3	2	0	0	0	26
1097.386	4	2	0	0	0	28
1138.412	3	3	0	0	0	32
1259.444	5	2	0	0	0	4
1300.468	4	3	0	0	0	2
1339.442	8	0	0	0	0	14
1341.497	3	4	0	0	0	1
1421.499	6	2	0	0	0	4
**1487.558**	3	4	0	0	1	4
1501.493	9	0	0	0	0	21
1503.545	4	4	0	0	0	4
1544.577	3	5	0	0	0	10
**1649.610**	4	4	0	0	1	5
1665.601	5	4	0	0	0	22
**1690.638**	3	5	0	0	1	1
1706.630	4	5	0	0	0	12
1747.650	3	6	0	0	0	68
**1811.661**	5	4	0	0	1	18
**1852.694**	4	5	0	0	1	10
**1893.714**	3	6	0	0	1	26

R/A: relative abundance.

Fucosylated species are reported in bold.

A main difference between HMOs and BMOs is the fact that most HMOs are highly fucosylated, while the presence of fucose in bovine milk has been controversial. Over 20 years ago Saito et al. [23] reported a single fucosylated oligosaccharide from bovine colostrum. Since that time numerous studies of BMOs have been undertaken and have revealed 40 oligosaccharide species and novel structural detail on BMOs; however no fucosylated species were described [16], [17], [24]–[28]. Indeed, the failure of numerous studies to identify fucosylated oligosaccharides in bovine milk led some to question [16] the original observations by Saito et al [23]. However, recently other studies reported the presence of fucosylated oligosaccharides in goat milk as well as bovine milk and colostrum [29]–[33]. For instance, Mariño et al. [29], employing fluorescent labeling of BMOs and their separation by hydrophilic interaction liquid chromatography (HILIC)-high performance liquid chromatography (HPLC), detected in bovine colostrum 2 fucosylated oligosaccharides: Fuc(α1–2)Gal(β1–4)Glc (2′-fucosyllactose) and GalNAc(α1–3)[Fuc(α1–2)]Gal(β1–4)Glc, the latter was previously reported in bovine [31] and goat colostrum [33]. However, Mariño et al. [29] did not detect 3-fucosyllactosamine, which was described in bovine colostrum by Saito et al. [23]. More recently oligosaccharides in bovine colostrum were also investigated by Aldredge et al [34], who employing nano-liquid chromatography tandem mass spectrometry, identified 5 fucosylated oligosaccharides that were found to be in common with human milk. These included 2′-fucosyllactose and 3′-fucosyllactose and 3 other oligosaccharides that were found to be in common with human milk, composed respectively of 2Hex1HexNAc1Fuc; 4Hex1HexNAc1Fuc; and, 3Hex2HexNAc1Fuc. In that work, bovine colostrum milk samples were collected from Jersey and Holstein cows within 12 h of calving. It is well known that bovine colostrum oligosaccharides composition is different from that of mature bovine milk [19] and it is reasonable to expect that it would be even more different in processed cheese whey obtained from late lactation milk. Furthermore, we hypothesize that the smaller oligosaccharides (including 2′-fucosyllactose and 3′-fucosyllactose) may have passed through the 1000 Da filtration membrane or even have co-crystallized with lactose during lactose crystallization in the production of mother liquor. These possibilities would explain why these oligosaccharides were not detected by mass spectrometric analysis in this work, whereas larger fucosylated oligosaccharides were identified.

Recently the bovine glycome was established for several dairy breeds using a combination of several high accuracy mass spectrometry instruments which revealed the presence of fucosylated oligosaccharides in Jersey breed [32].

The neutral HMOs (containing the monomers N-acetylglucosamine and fucose) are considered to be the most relevant factors for the development of the intestinal microbiota typical for breast-fed infants, whereas the acidic oligosaccharides (containing the monomer sialic acid) play an important role in the prevention of adhesion of pathogenic bacteria to the intestinal epithelial surface [13]. While specific glycans may act as decoys to inhibit binding of specific pathogens, the advantage of HMOs is likely due to the constellation of diverse glycan structures which act in concert to confer protection to infants from many bacterial, viral, fungal and other pathogens.

The present work, employing concentration techniques on dairy streams at pilot scale combined with advanced mass spectrometry, is the first to discover numerous high-molecular weight fucose-containing oligosaccharides in a whey stream of bovine milk. Fucosylated oligosaccharides were confirmed using accurate tandem mass spectrometry. The structures described herein likely evaded prior characterization because of their low abundance relative to the major BMOs. An analogous observation on human milk was reported by Finke et al. [4]. In that work, they reported that lactose and predominant oligosaccharides had to be removed by chromatography to allow the detection of minor high molecular weight oligosaccharides. In the present work, BMOs were fractionated and concentrated by membrane filtration and lactose was completely removed by solid-phase extraction prior to mass spectrometry.

The discovery of such a large number of fucose-containing oligosaccharides in bovine milk is promising for translation of these bioactive molecules into functional foods. HMOs containing fucose are associated with lower risk of diarrhea and respiratory diseases in breast-fed infants [35], [36]. Thus milk oligosaccharides, having structures analogous to cell surface receptors, may act as competitive inhibitors of pathogen binding to their glycoconjugate receptors. In particular, HMOs containing a 1,2-linked fucose inhibit the stable toxin-producing *Escherichia coli in vitro* and its toxin-induced secretory diarrhea *in vitro* and *in vivo,* and *Campylobacter jejuni in vitro* and *in vivo* [12], [35], [37].

## Conclusions

Mother liquor, a low-value by-product of whey and lactose manufacture, is a rich source of BMOs. Fractionation and concentration of these oligosaccharides by membrane filtration led to the production of a powder enriched in oligosaccharides and low in lactose. Structural analysis of the oligosaccharides present in this product through high-resolution and sensitive mass spectrometry revealed the presence of previously unreported high-molecular weight BMOs including some fucosylated structures.

Mother liquor is a better source of milk oligosaccharides than either mature bovine milk or colostrum. Extraction and enrichment or isolation of oligosaccharides from whey UF permeate or mother liquor can result in the development of biofunctional oligosaccharides ingredients for the food, beverage, and infant formula industries, add value to the waste streams of dairy production, and also reduce waste disposal costs for the dairy industry.

The presence of oligosaccharides containing GlcNAc, fucose and sialic acid make mother liquor potentially an ideal dairy source for commercial production of oligosaccharides-enriched ingredients for the food and beverage industries. When the bioactivities of the new molecules are investigated, not only would mother liquor reveal a broad spectrum of bioactivities beyond prebiotic activity, but also would lead to the production of value-added oligosaccharides ingredients and add value to the waste streams of whey and lactose manufacture.
